# The Importance of Predation Risk and Missed Opportunity Costs for Context-Dependent Foraging Patterns

**DOI:** 10.1371/journal.pone.0094107

**Published:** 2014-05-08

**Authors:** Jana A. Eccard, Thilo Liesenjohann

**Affiliations:** 1 Animal Ecology, University of Potsdam, Potsdam, Germany; 2 Animal Behaviour, University of Bielefeld, Bielefeld, Germany; 3 BioConsult, BioConsult SH GmbH, Husum, Germany; University of Western Australia, Australia

## Abstract

Correct assessment of risks and costs of foraging is vital for the fitness of foragers. Foragers should avoid predation risk and balance missed opportunities. In risk-heterogeneous landscapes animals prefer safer locations over riskier, constituting a landscape of fear. Risk-uniform landscapes do not offer this choice, all locations are equally risky. Here we investigate the effects of predation risk in patches, travelling risk between patches, and missed social opportunities on foraging decisions in risk-uniform and risk-heterogeous landscapes. We investigated patch leaving decisions of 20 common voles (*M. arvalis*) in three experimental landscapes: safe risk-uniform, risky risk-uniform and risk-heterogeneous. We varied both the predation risk level and the predation risk distribution between two patches experimentally and in steps, assuming that our manipulation consequently yield different distributions and levels of risk while foraging, risk while travelling, and costs of missed, social opportunities (MSOCs). We measured mean GUDs (giving-up density of food left in the patch) for both patches as a measure of foraging gain, and delta GUD, the differences among patches, as a measure of the spatial distribution of foraging effort over a period of six hours. Distribution of foraging effort was most even in the safe risk-uniform landscapes and least even in the risk-heterogeneous landscape, with risky risk-uniform landscapes in between. Foraging gain was higher in the safe than in the two riskier landscapes (both uniform and heterogeneous). Results supported predictions for the effects of risk in foraging patches and while travelling between patches, however predictions for the effects of missed social opportunities were not met in this short term experiment. Thus, both travelling and foraging risk contribute to distinct patterns observable high risk, risk-uniform landscapes.

## Introduction

Heterogeneous resource distributions and risks distributions over a landscape allow for individual differences in foraging behaviour [1] and therefore for the coexistence of species [2]–[4]. The distribution of resouces over landscapes and scales partly explains the distribution of its consumers (“foodscape” [5], [6]) which, in addition, have to reduce the risk of being consumed themselves. Foraging behaviour in risk heterogeneous environments has thus been a major focus of foraging ecology (e.g. [7]). Most studies confirm that foragers’ perception of predation risk (landscapes of fear, [8], [9]) strongly affects the resulting patterns of resource use, and foragers prefer safer food patches over riskier ones (for review [10]).

However, predation risk is not always distributed heterogeneously over a landscape. Risk can be uniformly distributed if hiding structures are missing, for example if predators are the same size and locomotion type as their prey [11] and prey cannot effectively hide. Further, risk can be uniformly distributed if structures are uniform in a scale relevant to the forager, as in modern agricultural landscapes and agricultural monocropping. If the predation risk by predators such as birds of prey cannot be localized for the forager, risk is perceived as ubiquitous and uniform. In experimental situations with risk-uniformity at high risk levels, we have observed a concentration of foraging effort on very few patches and a decline in foraging efficiency in these patches, both in artificial foraging landscapes [12], [13] and in real-world, risk-uniform landscapes [11]. Results were contradictory to predictions for patch use based only on distribution of resources, or on distribution of predation risk.

Patch leaving decisions in risk-uniformity may be explained with missed opportunities costs (MOC) of not foraging elsewhere or of not doing something else, i.e. the value an animal could achieve if spending its time on a different task. In most models and experiments, MOCs are considered to be equally high for all patches within an environment [14], but can differ among environments [15]. In environments with risk-uniform distribution at low predation risk, MOCs may be high because the value of other activities is high. Further, MOCs may also relate to seeking refuge. This hypothesis makes MOCs high at high risk levels, but refuges are not necessarily safe if the predator is of the same body size as the prey (e.g. weasels and voles, large ungulates and large predators). In this case, MOCs of not seeking refuge would therefore have a low value in high predation risk. In environments with risk-uniform distribution at high risk levels MOCs may be low, because also the conspecifics may avoid risks at high levels, remain inactive and are therefore unlikely to encounter. Therefore other activities besides foraging, such as socialising or mate search, may become less important.

Foragers may sample information about their environment in a *Bayesian* mode, increasing their knowledge about the environment and about MOCs step by step while foraging [15], [16]. A forager should therefore have higher MOCs if being not active, because the value of information is high. However, sampling is associated with higher predation risk. At an overall high predation risk, a forager thus has a limited knowledge about the value of currently visited patches compared to other patches because travelling is dangerous, and the forager may mis-value MOCs (which, on the other hand, increases the value of information in a risky landscape). Meanwhile, at an overall low risk, the forager can gather the necessary information and make more informed estimate of MOCs.

The above arguments illustrate how travelling risk, missed social opportunities, and predation risk can be entangled in patch leaving decisions. A joined investigation has hardly been addressed experimentally. Here we compare the foraging patterns of animals in a risk-heterogeneous landscape (RH) with patterns in safe landscapes with risk-uniform distribution of perceived predation risk (SRU) and in risky, risk-uniform landscapes (RRU). We assume different combinations for predation risk distribution, travelling risk and MOC distribution among the three types of landscapes (Table 1). In all landscapes, food distribution among patches shall be equal. With a reduced probability to encounter conspecifics (to socialise or to mate with), the value of travelling becomes lower and with it the MOC of not travelling. However, risk of travelling and MOCs are not always connected in the same direction: in a risk-heterogeneous landscape it may be risky to travel, but there are refuges to meet conspecifics, therefore so MOCc of not travelling remain high, despite its danger. We have therefore divided our predictions to predictions pertaining to travelling risk alone and predictions pertaining to MOCs of not being social, from here called costs of missed social opportunities MSOCs.

**Table 1:** Experimental manipulation of predation risk distribution at three different treatments in a 60 foraging experiment with 20 voles, Treatments: SRU safe, risk-uniform; RH risk-heterogeneity, RRU risky, risk-uniformity.

	treatments		
Manipulation	SRU	RH	RRU
**A) Assumed conditions**			
**perceived predation risk**	Uniform	**heterogeneous**	**uniform**
**perceived travelling risk**	**Lower**	higher	**higher**
**perceived MSOCs**	higher	higher	**lower**
**B) predictions for distribution of foraging effort (Δ GUD)**			
**by predation risk distribution**	lower	**higher**	**lower**
**by risk while travelling**	**lower**	higher	**higher**
**by MSOCs**	higher	higher	**lower**
**C) predictions for total foraging effort (Ø GUD)**			
**by predation risk distribution**	high	high	**low**
**by risk while travelling**	**high**	low	**low**
**by MSOCs**	high	high	**low**
**D) results: patch use by foraging animals (g millet/2 l sand)**			
Δ GUD	0.14±0.14	0.28±0.22	0.18±0.13
	**a**	**b**	ab
Ø GUD (low GUD = high effort)	**0.69±0.09**	0.80±0.12	0.80±0.10
	**a**	b	b
GUD at less exploited patch	0.76±0.12	0.94±0.07	0.89±0.10
	**a**	b	b
GUD at more exploited patch	0.63±0.12	0.66±0.14	0.71±0.14
	**a**	**a**	**a**

Assumptions, predictions and results, in fat print the treatment differing from the two others. GUD: giving-up-density of food left in the patch (mean +− standart deviation). Different small letters indicate significant difference among treatments in paired post-hoc comparisons, difference between b and (b): p<0.075.

We propose that based only on the distribution of predation risk among patches SRU and RRU should produce even distributions of foraging effort because patches do not differ in predation risk, while RH should produce an uneven distribution of foraging effort because animal prefer the safe patch over the unsafe (Table 1).

Travelling risk should be high both in the risky, uniform landscape RRU and the risk-heterogeneous landscape RH, while it is low in the safe, uniform landscape SRU. Therefore the difference among patches should be high in RRU and RH, and low in SRU (Table 1). Different predictions arise from MSOCs of other activities than foraging as an argument. If we assume, that animals can safely meet in the matrix or in refuges. MSOCs should be high in safe landscapes (SRU) and heterogeneous landscapes (RH). In risky landscapes (RRU) MSOCs are low, since focal forager and its conspecifics are forced to reduce their activity (Table 1). At the same time, the total amount foraged should be higher (i.e. low GUD) in the safe and heterogeneous landscapes (SRU and RH) since energy requirements of animals seeking conspecifics should be higher than in the risky landscapes (RRU). Resulting lower energy requirements are, however not separable from predictions due to predation risk in the patch. Predictions for energy gain in RH would vary with the assumption of whether or not the animal is aware of the fact that its experimental refuge is the only refuge available.

## Materials and Methods

### Animals

Common voles (*Microtus arvalis*, 12 males and 12 females) were kept under laboratory conditions 2–4 month prior to the experiment. Animals were captured by live trapping from premises of the University of Bielefeld, Germany (52°02’N, 8°29’E) and were returned after the experiment to the capture area. All animals were adult and sexually active. There was no difference in body weight between males and females (mean weight 22.9±4.2 g, t-test: t = −1.21, df = 22, p = 0.24).

### Ethical Note

For this study we documented the natural foraging behaviour of animals in a controlled environment. We do not consider the study as an animal experiment in the sense of the German Animal Protection Law (TierSchG §7(1)1) because it inflicted no pain, suffering or defects to the animal. There was no physical treatment, aggressive interaction, or risk of injury for the animals. Animal care and housing complied with institutional guidelines, permissions were given by the following authorities: Landesamt für Natur-, Umwelt- und Verbraucherschutz North-Rhine Westphalia (9.93.2.10.42.07.069 behavioural experiments, 360.12.06.01.3 capture of rodents from the wild), Gesundheits-, Veterinär- und Lebensmittelüberwachungsamt Bielefeld (530.42, keeping and breeding wild captured voles). Because of its non-invasive nature an approval for this particular study from the University of Bielefeld was not applied for.

### Experimental Set-up

**Figure 1:**
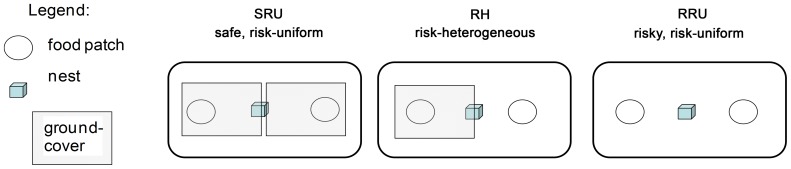
Experimental set-up for voles foraging in one of three artificial landscapes (4 m×2 m) with different risk levels and different spatial risk distributions. SRU: safe, risk-uniform landscape, food patches and nest were offered below a ground cover, RH: risk-heterogeneous landscape, one food patch was exposed, one was covered, RRU: risky, risk-uniform landscape with both food patches exposed.

Animals were kept singly in 8 outdoor arenas of 4 m×2 m, surrounded by a metal wall of 30 cm of height on a concrete floor, protected against bird intrusion by a 2.5 m high cage. The animals were housed in a shelter in the middle of the arena with a water bowl nearby the shelter, and were provided with two seed trays as foraging patches at either end of the elongate arena (Fig. 1). Seed trays measured 30 cm×20 cm×5 cm and contained 2 litres of sand mixed with 1 g millet seeds each. Experiments were run in three rounds, with 8 animals tested parallel per round.

Animals were habituated to the arenas for three days. We covered the floor and the feeding stations with wire mesh (mesh size 1 cm×1 cm) on 2 cm props. Animals touch the wire mesh with their backs and thus perceive a relative safety from potential avian predation while still able to perceive acoustic, visual and olfactory cues from the environment.

During habituation, seed trays were refilled with 2 g of dried millet every afternoon. Animals learned to exploit the trays overnight, and experienced that exploitation leads to diminishing returns over time. Patch leaving decisions should be based on the perceived level of risk and potential energy gains at alternative sources of food. Based on Browns extensions of Charnovs Marginal Value Theorem [14], [17] the trade-off between safety, foraging needs, and the importance of alternative opportunities is reflected in the exploitation levels of a food patch, the giving up density (GUD). It provides a reliable estimate of the perceived environment while foraging or travelling. With some of the factors contributing to GUD being equal, differences in GUD can be attributed to the remaining factor.

For the observation period we either removed the mesh cover and exposed both food patches and the shelter to simulate higher predation risk (risky, risk uniform (RRU) distribution of perceived predation risk), left the cover in place (safe, risk-uniform (SRU) distribution of perceived predation risk), or removed half of the cover, so that one patch was covered and one was exposed (risk-heterogeneous (RH), Fig. 1). Two additional treatments were established to investigate the trade-off between travelling and feeding among sexes including filming of foraging behaviour (Liesenjohann and Eccard, in revision), however these were not part of the comparison presented here. Each animal was subjected to each of the five treatments in a pseudo-random order. Not every animal used the food patches in every observation period, which reduced number of subjects from 8 to 4 animals after the first round. To maintain sample size, in later rounds we repeated failed treatments subsequent to the experiment, until each animal had used food patches (i.e. removed more than 0.05 g of food from at least one of the patches) during the observation period. The need for repetition was not associated to a particular treatment (chi2 = 2.6, df = 2, p = 0.260). We obtained complete data sets for 20 animals (10 males and 10 females). Together with habituation, animals stayed up to up to 2 weeks in the arenas.

We used the afternoon and evening hours (4–10 p.m.) as observation period since *M. arvalis* displays activity peaks before or after sunset [18]. The treatment conditions were established 18 h prior to the observation period (at 10 p.m. the previous day) with 2 g millet for the night in each tray to avoid delayed effects of previous treatments [13]. At 4.00 pm trays were refilled with 2 g millet each to start the observation period. In pilot studies we found this initial food density yielding enough accessible food for the animals but creating enough effort to harvest the food at the low depletion level, so that animals were not able to completely deplete a food patch during the 6 h period. After the observation period the sand from the trays was sifted, the remaining millet grains were dried and weighted, and treatment condition for the next day was established.

### Statistics

For each trial, we measured the Giving-up-density GUD (amount of millet in g per 2 l tray, that was left-over in the patch) from both patches. From this raw data we calculated mean GUD over both patches for estimates of total foraging gain (ø GUD), and the difference between GUDs among patches as a measure of distribution of foraging effort (Δ GUD). Multivariate ANOVA with a vector of the two dependent variables and ANOVAs with each of the separate variables were run in R [19] with the *car* package, always using animal ID as random factor and treatment as fixed factor. Paired post-hoc comparisons of main treatment effects within individuals based on these models were conducted using the Holm correction. Other statistics were computed with SPSS version 22 (IBM SPSS Statistics). Sex of the animal was initially used as a between-subject factor, but turned out to be non-significant and was removed from the reported models.

## Results

**Figure 2:**
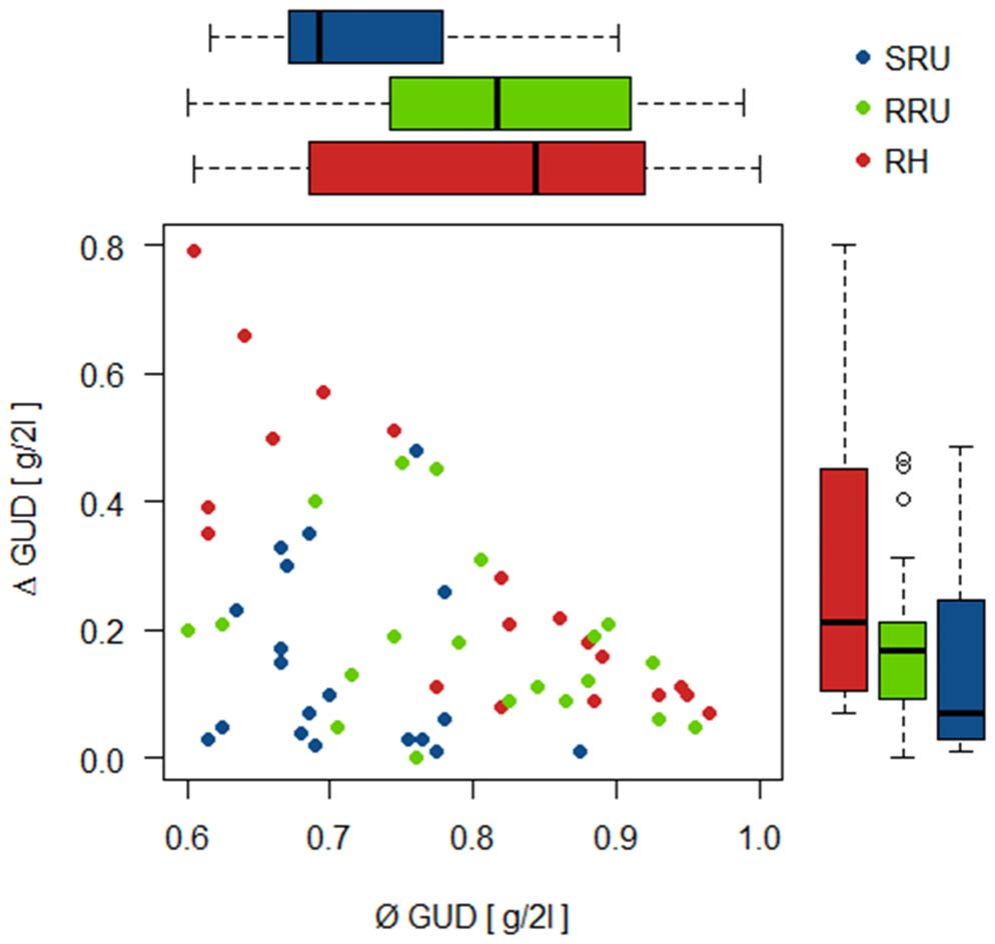
Mean giving-up density (GUD) of food from two trays, and the difference in GUD among the two trays in an experiment with 20 common voles in a two-patch choice situation. Find boxplots of the values in the three treatments parallel to the respective axes. Treatments SRU: safe, risk-uniform landscape, RH: risk-heterogeneous; RRU: risky, risk-uniform.

Over all trials (n = 59, with one trial removed from the analysis as an outlier, although it was supporting the results) animals produced mean GUDs (ø GUD) of 0.77 g +− 0.11 g over both patches (i.e. consumed a total of 0.48 g millet in 6 h) with a difference (Δ GUD) of 0.22 g +− 0.19 g among patches. Distribution Δ GUD and gain ø GUD were negatively correlated (Pearsons’s rho = −0,386, p = 0.003, n = 59, Fig. 2) over all trials, i.e. a higher consumption was generally produced by a more uneven consumption. This effect was due to the strong negative correlation and the large differences among trays, which were measured in the risk-heterogeneity treatment (RH: rho = −0.836, p<0.001, n = 20). Here the covered patch was always the most exploited by the animal. Variables were not correlated in the two risk-uniform treatments (SRU: rho = −0.141, p = 0.564, n = 19, RRU: rho = −0.326, p = 0.160, n = 20).

Both variables together differed among treatments (Multivariate ANOVA, fixed factor treatment: Wilks lambda = 0.484, F = 8.5, df = 4/78, p<0.001). Δ GUD was higher in risk-heterogeneity RH than in the two risk-uniformity treatments SRU and RRU (F = 4.9; df = 2/37; p = 0.0125; descriptives and post-hoc tests in Table 1 and 2). Ø GUD from both patches was lower in safe landscape SRU, compared to the two treatments including higher risk levels RH and RRU (F = 5.2; df = 2/37; p = 0.0099, Table 1). Looking at the raw data, this differences was due to the GUD at the less exploited patch (F = 23.70; df = 2/37; p<0.0001) being lower in the safe landscape SRU compared to RH and RRU and a tendency do differ between RH and RRU (Table 1 and 2) while GUD at the more exploited patch did not differ among treatments (F = 0.9; df = 2/37; p = 0.413 Table 1 and 2).

**Table 2:** Paired post-hoc test with HOLM correction in R, based on ANOVA models with individual as random factor.

Pairwise comparison	Δ GUD	Ø GUD	GUD at less exploited tray	GUD at more exploited tray
**SRU – RRU**	.292	.008	.001	0.29
**SRU - RH**	.025	.008	<.001	0.87
**RH - RRU**	.141	.949	.066	0.87

## Discussion

Both safe and risky risk-uniform landscapes SRU and RRU were similar in the distribution of foraging effort Δ GUD, but SRU had a higher total foraging gain (a low ø GUD, Fig. 2, Tab. 1 and 2) than RRU. The risk heterogeneous treatment RH was different from the safe RU treatment both in the more uneven distribution of effort and the lower total gain (i.e. higher GUD). RH was similar to the risky RU treatment both in low foraging gain and uneven Δ GUD distribution of foraging. Thus, Δ GUDs of the RRH treatment were inbetween those of SRU and RH. The results confirm predictions for the effects of travelling risk on total foraging effort, and for the effects of the distribution of predation risk among patches on the distribution of distribution of foraging effort. Predictions for MSOCs (lowest total and most uneven foraging effort in RRU) were not met (Table 1).

Foraging gains were equally low for RH and RRU, while animals harvested more food in the SRU treatment. Loosing foraging efficiency due to predation risk affects fitness negatively [20], however, if animals can expect risky conditions to be transitory, a reduction of foraging activity may be adaptive since losses can potentially be compensated at a later, less risky point in time (risk allocation hypothesis [21]). In our set-up we avoided delayed effects of previous risk treatments [13] by exposing the animals 18 h to the treatment before measuring its effects, so an initial reduction of activity should not be measureable during the observation period. The observed reduction in harvest rates both in RRH and RH may as well reflect the reduced energy needs of animals that have reduced their activity beside the absolutely necessary foraging. This would be expected for animals perceiving little opportunities, both foraging and social opportunities, outside the current foraging patch. Animals may thus be aware of the lack of social opportunities outside the refuge in our RH treatment, contrary to our initial predictions. Both the probability of surviving and the marginal value of time help comprising MOC [22] and also comprise our concept of MSOCs. However, for both variables the time scale is more long term (survivorship to next breeding season, value of time across longer scales) than what is experienced in the current patch and possibly in a 6-hour long experiment. Therefore longer experimentation and set-ups including conspecifics may be necessary to be able to measure MSOCs on foraging decisions.

Preference for safe foraging patches is a common result for risk-heterogeneous landscapes [10]. Although in this experiment the distribution of foraging effort Δ GUD did not differ among the two RU treatments, the GUDs of the less exploited patch were higher in RRU, supporting our earlier comparisons of more complex risk-uniform landscapes with different risk levels [12], [13].

Results indicate that if an alternative patch could be visited only under high risk, animals would neglect it. We here assume, that in a risk-heterogeneous landscape differences in MOCs are a by-product of predation risk distribution, while in a risk-uniform landscape an animal’s perception of MOCs develops while foraging. Such a *Bayesian* forager is gathering information along the foraging process [16], [22], [23]. In the patch already visited by the animals, the gains and risks of foraging are measurable for the animal, while at the yet unvisited patches risks and gains are difficult to estimate. MOCs to leave the known patch therefore assumed to be low by the animal. However, when risk is high, so is apprehension and foragers make mistakes in their foraging tasks. In RRH they may know less about the current patch because of high apprehension that they would know in a low risk situation, causing a greater spread in GUD among the two patches.

Conditions for risk-uniformity may be man-made in agricultural monocropping, but can also be natural, such as in predator-prey systems were the predator is of similar size and locomotive ability as the prey [11]. Our results show, that prey animals have adaptive foraging strategies also for risk-uniform landscapes. We suggest that risk-uniformity is an evolutionary effective environmental condition, and both predation risk and travelling risk are affecting foraging decisions in high risk, risk-uniform landscapes.

## References

[1] McIntyre NE, Wiens JA (1999) Interactions between landscape structure and animal behavior: the roles of heterogeneously distributed resources and food deprivation on movement patterns. Landscape Ecology 14: 437–447.

[2] Holt RD (1984) Spatial heterogeneity, indirect interactions, and the coexistence of prey species. American Naturalist 124: 377–406. 10.1086/284280 29519131

[3] Garb J, Kotler BP, Brown JS (2000) Foraging and community consequences of seed size for coexisting Negev Desert granivores. Oikos 88: 291–300.

[4] Bianchi F, Schellhorn NA, van der Werf W (2009) Foraging behaviour of predators in heterogeneous landscapes: the role of perceptual ability and diet breadth. Oikos 118: 1363–1372.

[5] Senft RL, Coughenour MB, Bailey DW, Rittenhouse LR, Sala OE, et al (1987) Large herbivore foraging and ecological hierarchies. Bioscience 37: 789–798.

[6] Searle KR, Stokes CJ, Gordon IJ (2008) When foraging and fear meet: using foraging hierarchies to inform assessments of landscapes of fear. Behavioral Ecology 19: 475–482.

[7] Abu Baker MA, Brown JS (2010) Islands of fear: effects of wooded patches on habitat suitability of the striped mouse in a South African grassland. Functional Ecology 24: 1313–1322.

[8] Laundre JW, Hernandez L, Altendorf KB (2001) Wolves, elk, and bison: reestablishing the “landscape of fear” in Yellowstone National Park, USA. Canadian Journal of Zoology-Revue Canadienne de Zoologie 79: 1401–1409.

[9] Brown JS, Laundre JW, Gurung M (1999) The ecology of fear: Optimal foraging, game theory, and trophic interactions. Journal of Mammalogy 80: 385–399.

[10] Verdolin JL (2006) Meta-analysis of foraging and predation risk trade-offs in terrestrial systems. Behavioral Ecology and Sociobiology 60: 457–464.

[11] Eccard JA, Pusenius J, Sundell J, Halle S, Ylönen H (2008) Foraging patterns of voles at heterogeneous avian and uniform mustelid predation risk. Oecologia 157 725–734. 10.1007/s00442-008-1100-4 18648858

[12] Eccard JA, Liesenjohann T (2008) Foraging decisions in risk-uniform landscapes. Plos One 3: e3438. 18927615 10.1371/journal.pone.0003438 PMC2562984

[13] Liesenjohann T, Eccard JA (2008) Foraging under uniform risk from different types of predators. BMC Ecology 8: 19. 19068146 10.1186/1472-6785-8-19 PMC2621151

[14] Brown JS (1988) Patch use as an indicator of habitat preference, predation risk, and competition. Behavioral Ecology and Sociobiology 22: 37–47.

[15] Olsson O, Holmgren NMA (1999) Gaining ecological information about Bayesian foragers through their behaviour. I. Models with predictions. Oikos 87: 251–263.

[16] Green RF (1980) Bayesian birds - a simple example of oaten stochastic-model of optimal foraging. Theoretical Population Biology 18: 244–256.

[17] Charnov EL (1976) Optimal foraging, marginal value theorem. Theoretical Population Biology 9: 129–136. 1273796 10.1016/0040-5809(76)90040-x

[18] Halle S, Lehmann U (1987) Circadian activity patterns, photoperiodic responses and population cycles in voles. Oecologia 71: 568–572. 28312229 10.1007/BF00379299

[19] Bujalska G (2000) The bank vole population in crabapple island. Polish Journal of Ecology 48: 97–106.

[20] Krause ET, Liesenjohann T (2012) Predation pressure and food abundance during early life alter risk-taking behaviour and growth of guppies (Poecilia reticulata). Behaviour 149: 1–14.

[21] Lima SL, Bednekoff PA (1999) Temporal variation in danger drives antipredator behavior: The predation risk allocation hypothesis. American Naturalist 153: 649–659. 10.1086/303202 29585647

[22] Olsson O (2006) Bayesian foraging with only two patch types. Oikos 112: 285–297.

[23] Olsson O, Molokwu MN (2007) On the missed opportunity cost, GUD, and estimating environmental quality. Israel Journal of Ecology & Evolution 53: 263–278.

